# UV Nanoimprint Lithography: Geometrical Impact on Filling Properties of Nanoscale Patterns

**DOI:** 10.3390/nano11030822

**Published:** 2021-03-23

**Authors:** Christine Thanner, Martin Eibelhuber

**Affiliations:** EV Group, DI Erich Thallner Str. 1, 4782 St. Florian am Inn, Austria; c.thanner@evgroup.com

**Keywords:** nanoimprint lithography, UV-NIL, SmartNIL

## Abstract

Ultraviolet (UV) Nanoimprint Lithography (NIL) is a replication method that is well known for its capability to address a wide range of pattern sizes and shapes. It has proven to be an efficient production method for patterning resist layers with features ranging from a few hundred micrometers and down to the nanometer range. Best results can be achieved if the fundamental behavior of the imprint resist and the pattern filling are considered by the equipment and process parameters. In particular, the material properties and pattern size and shape play a crucial role. For capillary force-driven filling behavior it is important to understand the influencing parameters and respective failure modes in order to optimize the processes for reliable full wafer manufacturing. In this work, the nanoimprint results obtained for different pattern geometries are compared with respect to pattern quality and residual layer thickness: The comprehensive overview of the relevant process parameters is helpful for setting up NIL processes for different nanostructures with minimum layer thickness.

## 1. Introduction

Since it was first mentioned in literature [1], nanoimprint lithography (NIL) emerged into an attractive patterning technique and developed considerably in terms of materials, process technology, and equipment. In the recent years, NIL technology has proven a wide range of capabilities, leading to its recommendation as a production suitable alternative lithography method. Particularly soft UV-NIL [2] is a very flexible and efficient technology applicable to a large variety of structure sizes and shapes [3], including complex shapes and 3D patterns [4] without compromising mass manufacturability.

Nowadays, UV-NIL is the method of choice for various emerging applications [5] due to the maturity of the technology (equipment, process, and materials) and its compatibility with semiconductor manufacturing environment. Even though NIL is still considered as niche technology compared to optical lithography, it already has a proven track record for volume production in fields like wafer level optics [6], augmented reality [7], and biomedical diagnostics [8]. In these areas, the key differentiator of this technology is the capability to pattern permanent functional layers and the ability to provide high-resolution patterns on large areas. These NIL benefits can overcome several limitations of other lithography techniques while being largely scalable in substrate size and production volume.

**Figure 1:**
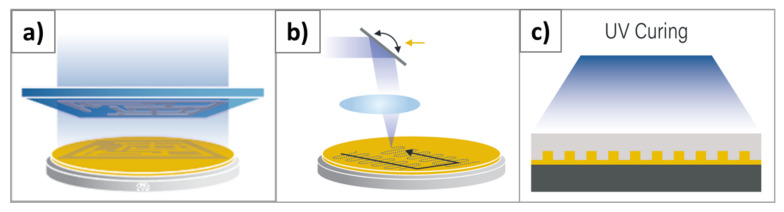
Schematic drawing of Ultraviolet (UV)-based lithographic techniques: (**a**) Optical lithography with a shadow mask, (**b**) direct writing, (**c**) UV-Nanoimprint Lithography (NIL).

However, as a replication technique, it behaves fundamentally different compared to lithography techniques based on (UV) exposure. As depicted in Figure 1a,b, those are typically based on the principle that only certain areas are exposed either by using shadow masks or by direct writing. For these areas, the UV exposure triggers a chemical reaction within the resist, and unexposed areas are not changed. The pattern can then be easily revealed during a development step that, depending on the resist type, will remove the exposed resist from the exposed areas for positive resists or unexposed areas for negative resist [9]. In contrast, UV-NIL, as shown in Figure 1c, is typically using UV exposure of all resist on the substrate and only negative resists are used. Additionally, most (optical) lithography is performed contactless, with a certain proximity gap between the mask and the resist, whereas it is obviously inherent to NIL process to contact the surface and fill the structures of the stamp used for patterning. This means that, while for other techniques it is most important to control the pattern quality during exposure by the optical system, in contrast, for NIL it is crucial to control the filling process.

The filling process has been extensively studied in previous work indicating to be a key contributor to certain failure modes as incomplete pattern replication, height, and residual layer variation [10,11]. Even though sometimes anticipated for spin-on UV-NIL, the results discussed by Sreenivasan and Cheng et al. refer to the high temperature and high-pressure regime of nanoimprint lithography by hot embossing. This type of filling is rather described as squeeze flow as elaborated by Rowland et al. [12]. The according defects in the presence of pattern and size variation for spin only thus have to be considered in this context as the shear resistance can cause various defects, including non-filled features and deformed features under pressure [10]. Other work such as that Song et al. [13,14] consider the filling in a low-pressure regime using a method referred as reverse NIL. In this case, the stamp is coated, and the patterns are transferred under pressure and heat afterwards. Consequently the filling and the pattern transfer are separated and not directly comparable to a to a conventual UV-NIL process.

For the UV-NIL, numerical descriptions as done by Taylor et al [15,16,17,18] and Yin et al. [19,20] take the next external forces as well as the capillary forces and the according surface energies into account, and thus seem to be the most applicable. However, some experimental findings like lateral redistribution of low viscous spin on resist and according changes in filling behavior are not fully explained. Particularly, defects occurring close to critical resist thickness for minimum residual layers cannot be fully explained. Previous work from Lee et al. [21] and Yasuda et al. [22] indicate that the filling was mainly investigated in a pressure-controlled regime rather than in the capillary regime. As will be shown in this work, state-of-the-art NIL stamp and resist combinations appear to have different surface energies and the filling is rather dominated by the fluidic properties.

Consequently, NIL processes have different prerequisites to achieve high quality patterning and it is essential to understand the influencing parameters and boundary conditions for reliable manufacturing. This work focuses on detailed analysis of the post-imprint residual layer, resist viscosity, and its flowing behavior (redistribution) as well as their respective impact on the filling behavior of various shapes of nanostructures.

## 2. Materials and Methods

**Figure 2:**
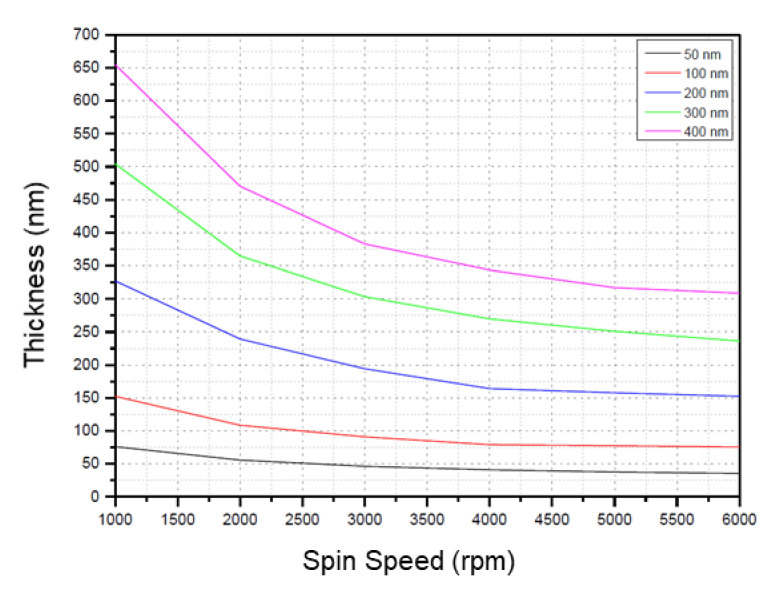
Spin curves of EVG UV-NIL AS with different solvent dilution. The solvent content is optimized for different thickness at 2500 rpm and 60 s spin time. The respective thickness values are shown in the inset.

The study is based on the EVG SmartNIL technology using an EVG7200 imprint system (EV Group, St. Florian am Inn, Austria). All imprints were done on full 8” wafers, which were spin coated on an EVG101 coating system (EV Group, St. Florian am Inn, Austria). Two different resist materials have been used. EVG UV-NIL AS (EV Group, St. Florian am Inn, Austria) is used for the different geometric structures and was chosen for those tests as it shows typically very good filling behavior. Additionally, the layer thickness control can be easily achieved by dedicated versions optimized for different thickness at 2500 rpm and 60 s spin time as shown in Figure 2. For thicknesses below 50 nm, the solvent content has been manually adjusted to achieve a final thickness of only 20 nm.

The according spin speeds for the used thicknesses of 90 nm, 55 nm, and 20 nm were 2800 rpm, 1900 rpm, and 2000 rpm, respectively, for 1 min. All coatings were followed by a solvent release bake on a hot plate at 120 °C for 1 min.

For the second part of the study EVG UV-NIL E has been used as it was found that the viscosity could be also measured Tuning Fork Vibro Viscometer without additional solvent. The viscometer uses 2 mL samples to measure a viscosity range from 0.3–1000 cP (mPa⋅s). To have a solvent free reference is important for better understanding of the fluidic behavior as during the actual imprint all resists are considered solvent free due to the soft bake.

Both resists can be imprinted under comparable process conditions. Standard parameter at room temperature are 0 s delay time after full contact and exposure for 30 s at 300 mW/cm² from a 365 nm LED light source.

Additionally, specific UV-NIL working stamps have been used. The EVG NIL UV/AF working stamp material can be spin-coated and UV cured like other resins and does not need any additional treatment. The coating parameter were 3500 rpm for 5 min without any soft bake. To make the working stamp, the material was cured for 200 s with 300 mW/cm² at 365 nm.

For the characterization of the imprinted structures the Brooker Nanoscope AFM system has mainly been used. In detail, FASTSCAN C needles with 5 nm tip radius were used in Peak Force Tapping mode. Typical scan fields were 10 × 10 µm to 20 × 20 µm. For the investigation of larger areas brightfield images were made by a Zeiss Axiotron optical microscope with 5× to 50× magnification.

Additionally, to determine the viscosity of the EVG UV-NIL E resist, a Brookfield DV2TLV rotating viscometer was used. It allows to measure a viscosity range 15–30,000 mPa⋅s with a temperature sensing range of −100 °C to 300 °C. For the experiments, only a range from room temperature to 60 °C has been considered to determine the viscosity vs. the temperature.

## 3. Results

**Figure 3:**
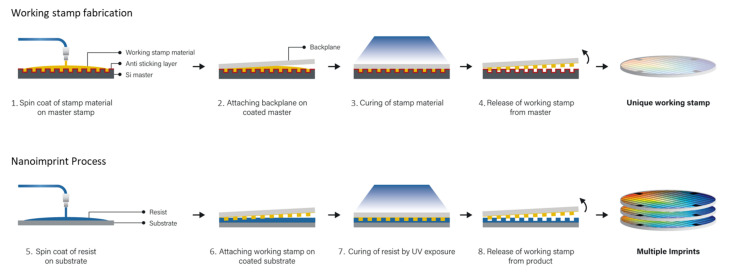
Schematic illustration of the SmartNIL process flow. Steps 1–4 show the typical manufacturing steps for creating a transparent working stamp. Steps 5–8 illustrate the actual imprint process which can be repeated multiple times.

For the study of the UV-Nanoimprint process, the SmartNIL^®^ technology has been used. This is a UV-NIL method performing a wafer level or full substrate imprint using transparent and flexible polymer working stamps. The complete wafer level process flow is shown in Figure 3. This includes the manufacturing of the working stamp (steps 1–4) and the actual imprint process (steps 5–8) [23,24]. As a starting point, for each imprinted pattern, there has to be a master stamp manufactured. The master stamp provides the actual topography of the desired structures for the replication and is most commonly, in particular for nanostructures, prepared on silicon using e-beam lithography and subsequent etching. However, for the imprint process, the actual patterning method and material are secondary if they are compatible with the materials used for replicating the working stamp. The replication process is using a working stamp, which is produced based on the master stamp. The working stamp fabrication starts with the coating of an anti-sticking layer (ASL) applied to the master stamp: This significantly increases the contact angle of the surface and minimizes the surface energy for the working stamp material, resulting in improved detaching of the working stamp after production. Secondly, the working stamp material is dispensed by spin coating and a flexible backplane is mounted. After contact with the master stamp the working stamp polymer material is cured and the negative of the master stamp surface topography is replicated in the working stamp polymer. Finally, the working stamp can be demolded after solidification, and is then used for the actual nanoimprint process.

For nanoimprinting and effectively patterning the target substrate, the nanoimprint resist is spin coated onto the respective surface and a soft bake is performed. The resist thickness and uniformity are crucial for the filling of the structures: This also has to consider the aspect ratio and fill factor of the master design. Subsequently, the flexible working stamp is fully conformal applied to the substrate, which is enabled by the flexible backplane support. While the working stamp and the wafer surface are in contact, the stamp structures are filled by capillary forces. The key parameters for this filling are further investigated in this paper: The amount of resist, the resist viscosity, and the flow behavior. Those parameters have significant impact on the residual layer thickness and the replication quality in terms of pattern fidelity and defects. Finally, the resist is cured under UV exposure and the working stamp can be demolded. Multiple imprints can be performed with a single working stamp as it can be reused.

Even though the above-discussed full SmartNIL imprint process is split in two parts of manufacturing the working stamp and subsequently imprinting the wafer, the entire process can be implemented on the same equipment. This work was performed on the EVG^®^101 spin coating and the EVG^®^7200 SmartNIL system using 200 mm wafers.

**Figure 4:**
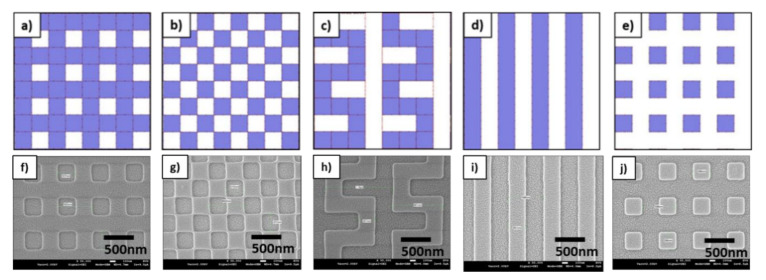
Schematic illustration of the different geometrical layouts used to investigate the filling behavior of nanostructures: (**a**) Crossbar, (**b**) checkerboard, (**c**) meanders, (**d**) line and space (L/S), and (**e**) pillars. Pattern dimensions of 300 nm with a depth of 110 nm have been selected for comparison and respective SEM images of SmartNIL replications are shown from (**f**–**j**).

In order to study the filling properties based on the available imprint resist material, viscosity, and flow behavior, an e-beam written and etched silicon master was used. On this master, several arrays with different structure geometries as described in Figure 4a–e are present. The pattern investigated pattern sizes were 300 nm with a depth of approximately 110 nm have been replicated by SmartNIL and according SEM images are shown in Figure 4f–j.

For imprint processes it is very often crucial to control the layer uniformity and resist thickness in order to achieve the desired imprint quality. In particular, this is required if the residual layer has to be minimized in order to facilitate subsequent etching processes using the imprinted features as mask. Therefore, the impact of the resist thickness on the filling behavior and the resulting residual layer has been investigated in more detail.

**Figure 5:**
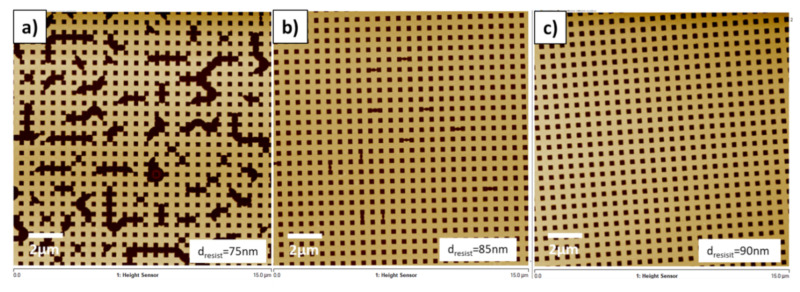
AFM images of the imprinted crossbar pattern using different resist layer thickness: (**a**) Fully replicated pattern with enough resist volume (d_resist_ = 90 nm), (**b**) Imprint wits slightly less resist volume showing minor defects (d_resist_ = 85 nm), and (**c**) Imprint with too less resist volume showing significant defects (d_resist_ = 75 nm).

At first, a crossbar structure was investigated with fill factor of 75% and all connected patterns. From the theoretical calculations according to formula d_resist_ = ((pitch)^2^ − (width)^2^) × depth/(pitch)^2^, the ideal resist thickness for minimum residual layer is considered 82.5 nm. The same value can be estimated by just using 75% of the pattern. Practically, as it is not possible to achieve zero residual layer with typical imprint resists, the actual resist thickness should be chosen at least a few nm above the ideal value. As shown in the Atomic Force Microscope (AFM) images in Figure 5, the imprint process was performed with different resist thickness: 90 nm, 85 nm, and 75 nm, respectively. In order to achieve the different layer thickness, a single resist type (EVG UV-NIL A) was used, but the spin coating parameters were varied. For 90 nm layer thickness was used a speed of 1500 rpm for 60 s, while for 85 nm and 75 nm, 1800 rpm and 2500 rpm were used, respectively.

In this way the amount of the resist was heuristically lowered to achieve a minimum residual layer and was further reduced to a level where the filling of the imprint pattern was limited by the available resist volume. Working intuitively is often wrong considering that this will rather impact the vertical filling of the structures. This is only true for macroscopic structures where the external applied printing force is domination the filling behavior but cannot be extrapolated to the nanoscale region. For these structures, capillary forces start to dominate the process [10] and change the filling behavior significantly. Therefore, Figure 5 shows imprints where the resist layer was set to be (a) slightly more, (b) approximately equal to, and (c) less than required filling volume. In detail it is observable in Figure 5a that for the 300 nm crossbar pattern a complete filling of the structures could be achieved with a 90 nm thick resist layer. When lowering the resist thickness to 85 nm as shown in Figure 5b, minor defects were occurring randomly. Those defects significantly increase when the resist layer thickness is further decreased to 75 nm. This shows that the nanostructures will be filled in some areas completely while in other areas the resist is completely missing. Thus, it is concluded that the resist volume will be redistributed and the capillary forces dominate the filling behavior.

**Figure 6:**
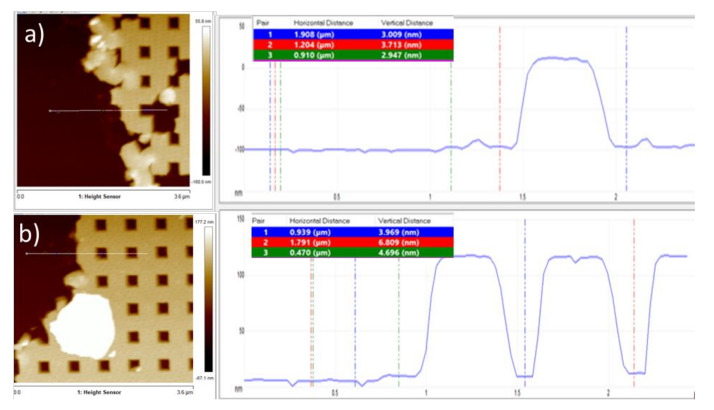
Atomic Force Microscope (AFM) top view image and height profile of the imprinted crossbar pattern used to measure the residual layer thickness for: (**a**) Complete filling (d_resist_ = 90 nm) and (**b**) incomplete filling (d_resist_ = 75 nm).

Next to the filling of the stamp structure it is also important to understand the impact on the residual layer control. The residual layer thickness has been determined: part of the imprinted structure was removed by scratching down to substrate surface and the step created was investigated using AFM measurements. Figure 6a,b show the topography measured for the 90 nm and respectively 75 nm samples. The measurement is considering that no resist is left for the scratched areas and the thickness is measured from the substrate surface. The residual layer thickness is defined by the height difference to the valleys between the crossbar structures. In this way for the completely filled 90 nm structure an average residual layer thickness of about 5 nm was observed. In comparison the 75 nm sample the average residual layer thickness is about 3 nm. Interestingly, this seems also to be valid for areas with missing structures, where it could be assumed that all the resist was transferred to the filled areas. Even though this finding has to be verified in more detail with different imprint resist, it clearly shows the limitations of residual layer control in the <10 nm range and additionally confirms that with conventional materials and processes a residual layer free imprinting cannot be achieved.

Notably, also a second aspect indicated above could be approved by this measurement. It was assumed from the top view measurements above the full pattern height achieved for the 75 nm layer thickness by redistribution of the resist. As can be seen in the AFM topography data the pattern height of 110 nm is similar to the completely filled sample with 90 nm layer thickness. This proofs that the tendency to fill the topography rather completely is more pronounced than keeping a uniform layer beneath the pattern and fill them only partly in height as observed in the pressure regime [17,18]. Thus it is concluded the dominating nature for the structure filling of the nanostructures is given by the capillary regime.

**Figure 7:**
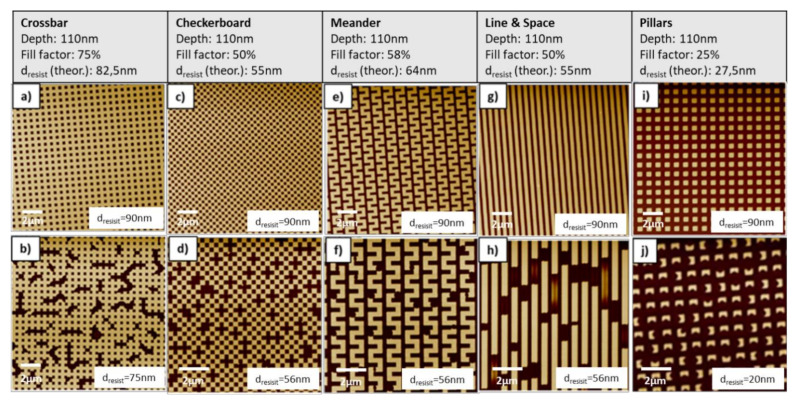
Top view images showing a comparison between different geometries after imprinting: (**a**,**b**) Crossbar, (**c**,**d**) checkerboard, (**e**,**f)** meanders, (**g**,**h**) line and space (L/S), and (**i**,**j**) pillars. Top row shows the fully replicated patterns using optimum resist volume. Bottom row shows the observed defects if less than optimum resist volume is used. The critical dimension for all patterns geometries was 300 nm.

As the observed behavior might be linked to the actual structure design, the impact on different pattern shapes has been investigated as well (Figure 7). As the different structures do not have the same filling factor, the resist layer thickness has been modified for the different designs. The crossbar design already discussed in Figure 5 and Figure 6 has a fill factor of 83% and the structures are all connected. When comparing Figure 7a of the fully replicated structure with Figure 7b of the partially filled structure it seems that the geometry also influences the defect pattern. When the amount of resist is lowered below the optimum these defects occur more or less at random positions but overall rather uniformly distributed. The defects seem to be mostly connected with the holes in the crossbar pattern and interacting with them but often with less defined roundish borders. Additionally, it appears that the non-filled area also rather tends to expand and connect, resulting in large non-patterned areas with sharp borders to fully patterned, non-defective areas.

In order to investigate the impact of other pattern geometries independently from the fill factor, three different designs with the same critical dimension of 300 nm and comparable fill factor have been analyzed. These are the checkerboard with a fill factor of 50%, the meander structure with a fill factor of 58%, and the L/S structure with 50% fill factor. For these structures, the resist thickness had to be lowered until incomplete filling appeared. For all three structures, a critical resists layer thickness of 56 nm was observed.

The checkerboard structure shown in Figure 7c,d is geometrically the most similar to the crossbar structure as it has similar holes, and the pattern area is still quasi connected through the edges of the pattern. As can be seen in Figure 7d, in this case, the defects are randomly but quite uniformly distributed. However, as the resist cannot freely flow, the defects are mainly complete missing patterns with well-defined borders, which appear to be more isolated.

Figure 7e,f show the impact on the meander structures: This geometry is still relatively close to the crossbar pattern but in this case, the valleys are also connected over a larger area. As it can be seen in Figure 7f, the defects are significantly different as compared to Figure 7d. While defects of the checkerboard in Figure 7d tended to be square-shaped, in contrast to the meander structure in Figure 7f, the tendency of the defects is clearly to be more rounded. This rounded shape is independent if the pattern starts at a corner or in the middle of a meander line and indicates that the defects start from a single point and have isotropic behavior if not influenced by the pattern. The heuristic comparison of the behavior of the checkerboard and the meander structures to the crossbar pattern shows a good overlap.

The change of the structure to L/S as shown in Figure 7g,h (50% filling factor) limits the redistribution of the resist in 1 dimension. As it can be seen also for this case, the incomplete filled structures show defects also only along a single direction. Specifically, it creates intersected lines with completely filled and unfilled parts. Interestingly, there are also some sections where the lines appear to be partly filled in height. Even though this seems not to be the dominating effect, it can be clearly seen that this intermediate filling exists as well. Even though not completely verified during this study, it is assumed that for these areas, a metastable state was fixed during curing. Further tests would be needed to verify if, with longer filling time or lower viscosity, these parts would have merged with already fully filled areas as well. Alternatively, it could also indicate a minimum line length to achieve complete filling.

Finally, the impact of too-thin resist layers was investigated for the inverse geometry of the crossbar structure in Figure 7i,j. The filling factor of this structure is only 25% and the according critical resist thickness is 27.5 nm. Additionally, there are no connections between the patterns that could support the redistribution of the resist. As for all other patterns studied in this series, it can be clearly seen that complete filling and a good pattern replication was also possible for the pillar structure with a 90 nm thick resist layer. To achieve the critical regime with clearly observable defects, the resist thickness was lowered to only 20 nm. As shown in Figure 7j, for these isolated structures it was not possible to redistribute the resist from one pattern to the other and no completely missing structures were observed. Consequently, to compensate for the missing resist volume every single pillar shows a small defect. This is quite remarkable, that even on this small area a resist redistribution similar as for the other patterns could be shown. Due to the capillary filling behavior, it is often experienced that small pillars rather get stuck in the stamp as there is no strong adhesion to the surface via the residual layer given anymore.

**Figure 8:**
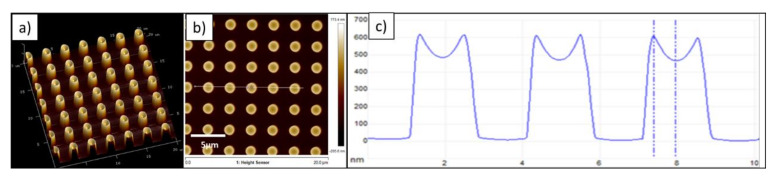
AFM images showing incomplete filling behavior of pillars with 1 µm diameter and 1.3 µm height. (**a**) Tilted view, (**b**) top view, and (**c**) height profile. In the top view, the line scan for the height profile can be seen.

To understand if this effect is still present for larger pillars, a master with a structure of round pillars was replicated. The diameter of the pillars was 1 µm, with a height of 1.3 µm. The AFM images in Figure 8 show clearly a different behavior when compared to the 300 nm pillars with 110 nm depth. The AFM top view image in Figure 8a shows that for this pattern, no defects occur if the resist layer is too thin. However, as the volumes of the master and the offered resist must match, a different type of filling must occur and thus the structures will not be filled to the top. This fact is confirmed by the results shown in Figure 8b,c, where the structure height of about 620 nm is approximately half of the depth provided by the master template. All the pillars have about the same height and are approximately half filled. In addition, a distinct dimple in the center of every pillar is observed. This exactly fits to the expectations for capillary filling in case of macroscopic structures.

**Figure 9:**
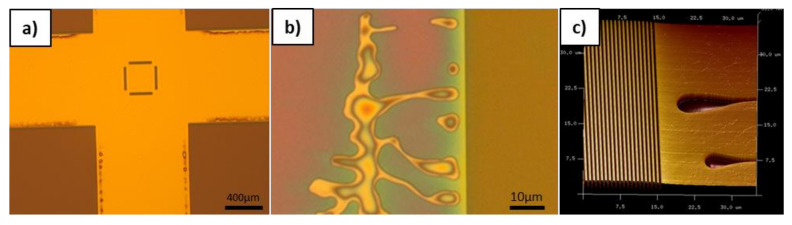
(**a**) Optical microscope image of imprinted L/S arrays (dark) and non-pattered areas (bright), (**b**) detail optical microscope image of defects occurring due to too low resist volume, and (**c**) AFM image of the imprinted L/S pattern showing the neighboring defects.

As in the above study, a lateral redistribution of the resist was observed within the pattern area, it was also of interest to investigate impact on the surrounding area. Therefore, a master with 400 nm L/S pattern with fields separated by about 1 mm non-patterned area was chosen. Within the pattern area, there is a uniform 50% fill factor but at the border there is a change of the fill factor to 100%. It is considered that in this area, the lateral flow of the resist is not hindered by the pattern and thus shows approximately the free path length of the resist redistribution. As it can be observed in the optical microscope images in Figure 9a and according to the detail picture in Figure 9b, there is a large lateral redistribution of the resist. While the dark areas with the L/S pattern are homogenously filled, it can be clearly seen that at the border of these structures in the non-patterned bright area large defects occurred. In the detailed image in Figure 9b, it is clearly observable that there is a redistribution of the resist within a range of several tens of micrometers. The AFM image in Figure 9c confirms this redistribution while showing defect areas next to the completely filled L/S patterns. Interestingly, the shape of the defects is comparable to an elongated droplet like structure. Generally, the shape and the distance of the defects show some unexpected behavior. There are no defects directly at the border, but some are present in the border vicinity. However, the largest defects are just a few tens of micrometers away from the patterned area. This is yet unclear and might be intermixed with other effects not only based on the lateral redistribution of the resist to fill the patterned area. However, it could be proven that in non-patterned areas there is a long-range effect on the filling of the structures. This also implies that the lateral flow of the resist will also be for patterned areas will be on longer range until the required resist volume is consumed.

**Figure 10:**
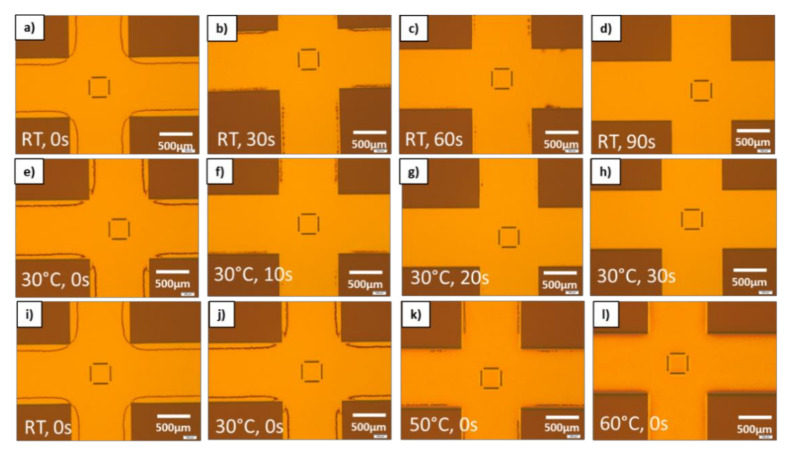
Filling behavior matrix for different temperatures and delay times until the defects completely. (**a**–**d**) Constant room temperature and increasing delay time in 30 s steps. (**e**–**h**) Constant elevated temperature at 30 °C and delay time increase by 10 s steps. (**i**–**l**) Constant delay time of 0 s and increasing temperature from RT up to 60 °C.

Obviously, it is not desired to have such defects in areas without patterns as well and thus it requires process optimizations to overcome this issue. As it is indicated that this is again strongly related to the fluidic properties, two key process parameters have been investigated on its effect on the border defects. As there are no structures where the defects occur it is obvious the capillary forces of the nanostructures are dominantly gaining material from this area. Even though there is enough material available in the unpatterned area, the defects are not compensated immediately if there is no delay time between full contact and UV exposure. For this process conditions, the defect area is well pronounced all around the edge zones of the patterns in a distinct distance from the L/S structure as depicted in Figure 10a. Adding delay time allows the material to redistribute better and to close the defects. It is easily observable in Figure 10a–d that the defects are vanishing more and more with increasing delay time from 30 s–90 s. At delay time of 90 s the defected zone is completely gone, showing that this effect is clearly related fluidic properties of the material. In Figure 10e–h it could be shown that by increasing the temperature to 30 °C the filling times could be reduced significantly. This is indicating the expected viscosity change of the resist at elevated temperatures. Thus, in a third test series, the viscosity of the resist was further lowered by increasing the temperature systematically without adding delay time. For all temperature dependent tests, a heated chuck was used, which can reach temperatures up to 60 °C. In Figure 10i,l,h, it is shown that increased temperature has a comparable influence as the delay time. The defects are strongly reduced by lowering the viscosity and fully vanished at 60 °C. This clearly proves that the resist redistribution is strongly liked to given filling and resist viscosity.

**Figure 11:**
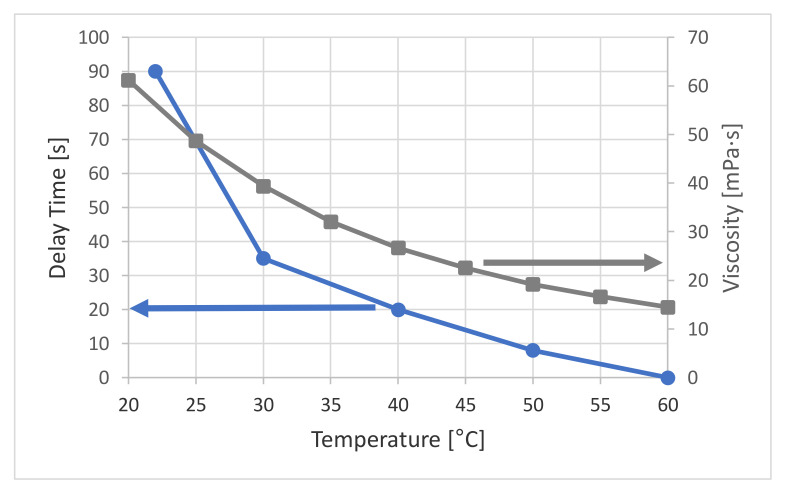
(**Left**) Plot of the required delay time versus the imprint temperature for defect free replication. (**Right**) Temperature dependence of the resist viscosity.

The dependence between delay time and imprint temperature is plotted in Figure 11 showing all series that have been investigated. The blue data series shows the time needed to have ideal imprint at a certain temperature. It is significantly dropping with increased temperature. This behavior correlates very well with the viscosity change measured within temperature range using a viscosimeter. Thus, for setting up NIL processes particularly with less known materials, temperature can be a crucial parameter to optimize the process and to enable proper replication and filling to the structures.

Overall, it could be demonstrated that the imprint process with state of the art material combinations are rather driven by capillary forces and not pressure driven as often found and discussed in earlier work [10,11,12] Thus, the fluidic properties, surface energies, and according capillary forces have a significant impact on the imprinting process and have to be taken more into account for the description of the UV-NIL process. Even though the capillary regime has been numerically described by Taylor et al. [13,14,15,16] lateral redistribution is mainly discussed in context of droplet spreading for layers dispensed by inkjet coating and laterally confined stamps used for step and repeat NIL [15,25]. According to the observations in this work, the lateral redistribution of full area UV-NIL imprints should be considered differently. It seems that those step and repeat approach is still best understood in the pressure regime and the main criteria for redistribution is the droplet spreading [15]. Wafer level processes like SmartNIL use flexible stamps and thus apply only comparably low force. Thus, state-of-the-art imprint materials are designed to work best in the capillary force dominated regime. Additionally, due to the much larger dimensions full area NIL takes more time until full area contact. This will allow certain redistribution also during the contact phase. Consequently, even without delay time between full contact and exposure this effect has been observed more easily. In particular, when aiming for a minimum residual layer, a critical resist thickness can be observed where lateral effects are strongly influencing the process control. An additional contrast is given by the fact that the spin coated areas can provide an additional resist reservoir for lateral redistribution at the boarders of the pattern areas. As described in this work, depending on the resist viscosity and the delay time, significant resist redistribution has to be considered on length scales of tens of µm. This shows the fluidic properties of the resist and the capillary forces are also crucial if there are major local changes of the patterns and according filling factors. If the viscosity or the given time are too low it is likely that defects with missing resist in those redistribution zones can occur. Therefore, improved process conditions with increased delay times or reduced viscosity can help to overcome this. Generally, it could be proven that low force techniques like SmartNIL, which support the inherent filling behavior of the resist, are very well suited to lower the layer thickness to critical condition and allow to achieve very thin layer imprints with high residual layer control. Temperature-controlled chucks can further improve the filling behavior to optimize the process conditions for high-quality imprints.

## 4. Conclusions

The study of minimizing the resist thickness even below the volume requirement for complete replication of the structures proves the filling of the structures is strongly dependent on fluidic properties of the resist and the capillary forces. This is of particular interest when the provided resist volume is at the limits for completely filling the structures. It is also clearly shown that at the nanoscale significant redistribution of the resist is present without external force. This general behavior is observed by filling studies showing comparable effects for all investigated structure geometries. The larger the connected areas, the more pronounced the lateral redistribution of the resist is. Connected structures thus tend to have more extended defects while isolated structures have a defect on every pattern. However, this behavior is only true if the geometrical dimensions are below a certain limit. For larger structures, there is no lateral redistribution and the structures get only shallower but still showing typical indications for capillary filling.

Additionally, it could be shown the capillary forces have a significant and long-range impact also in lateral direction. For areas with significant changes of the pattern density, edge effects occur showing evidence that resist is flowing from one to the other area by leaving defects in these zones. These flow defects can be reduced or completely avoided by prolonged delay times or reduced resist viscosity by using a heated chuck.

Summarizing, this has study shown that the fluidic properties of the resist and the pattern geometry have to be considered to set up reliable manufacturing processes. This is particularly to be considered for nanoscale structures with very thin residual layers, and the filling behavior can deviate significantly from expectations based on macroscopic experience.

## Data Availability

Data is contained within the article or supplementary materials.
